# Effects of Parasitism and Morphology on Squirrelpox Virus Seroprevalence in Grey Squirrels (*Sciurus carolinensis*)

**DOI:** 10.1371/journal.pone.0083106

**Published:** 2014-01-08

**Authors:** Natasha E. McGowan, Nikki J. Marks, Colin J. McInnes, David Deane, Aaron G. Maule, Michael Scantlebury

**Affiliations:** 1 School of Biological Sciences, Queen's University Belfast, Belfast, United Kingdom; 2 Vaccines and Diagnostics, Moredun Research Institute, Edinburgh, United Kingdom; University of Milan-Bicocca, Italy

## Abstract

Invasive species have been cited as major causes of population extinctions in several animal and plant classes worldwide. The North American grey squirrel (*Sciurus carolinensis*) has a major detrimental effect on native red squirrel (*Sciurus vulgaris*) populations across Britain and Ireland, in part because it can be a reservoir host for the deadly squirrelpox virus (SQPV). Whilst various researchers have investigated the epizootiology of SQPV disease in grey squirrels and have modelled the consequent effects on red squirrel populations, less work has examined morphological and physiological characteristics that might make individual grey squirrels more susceptible to contracting SQPV. The current study investigated the putative relationships between morphology, parasitism, and SQPV exposure in grey squirrels. We found geographical, sex, and morphological differences in SQPV seroprevalence. In particular, larger animals, those with wide zygomatic arch widths (ZAW), males with large testes, and individuals with concurrent nematode and/or coccidial infections had an increased seroprevalence of SQPV. In addition, males with larger spleens, particularly those with narrow ZAW, were more likely to be exposed to SQPV. Overall these results show that there is variation in SQPV seroprevalence in grey squirrels and that, consequently, certain individual, or populations of, grey squirrels might be more responsible for transmitting SQPV to native red squirrel populations.

## Introduction

There is a strong link between infectious disease and biodiversity because individual success, survival, and, consequently, the viability of entire host populations depend upon interactions with external pathogens [1]. The effects of infection are related to a number of factors including pathogen virulence [2], the nutritional and reproductive status of the host [3], [4], as well as various biotic and abiotic factors such as ambient temperature and intraspecific competition [5], [6]. Virulent pathogens may result in the rapid death of their host and initiate subsequent host population decline [7], whereas others can persist as low-grade chronic infections that are mediated immunologically [8]. The pathology associated with chronic disease may be important, being manifest as reductions in host growth, longevity and/or fecundity [1]. In addition, chronically diseased animals can be “carriers” within an ecosystem, potentially transmitting infection to other individuals [9]. The arrival of novel pathogens and/or infected hosts/vectors into a geographical area where the resident species were not previously exposed could facilitate disease transmission [10]. Diseases in resident individuals may then present as acute rather than chronic [11].

Many impacts of disease on host behaviour and life history are mediated through modifications in host energy expenditure [12]. Investment in one aspect of somatic function, such as fighting an infection, inevitably means that those resources cannot be invested in others such as reproduction or growth [13]. Conversely, the success of a host population depends upon an adequate supply of food from the environment such that reductions in food availability can increase the probability of infection [1]. For example, sub-lethal effects of a viral pathogen infecting Indian meal moths, *Plodia interpunctella*, were more apparent when food was limited [4]. As food availability within an environment may fluctuate over time, e.g. seasonally, the prevalence and impacts of infectious disease may also coincide with such fluctuations. Finally, the effects of infection may be exacerbated by the presence of multiple pathogens, such that animals harbouring one pathogen may be more susceptible to, or suffer greater effects from a secondary infection. This may be a consequence of a diversion of the host's resources towards the primary infection, thereby allowing the secondary pathogen to evade the immune system [14], [15]. For example, myxoma virus correlates with nematode and cestode burden in the European rabbit (*Oryctolagus cuniculus*), which was suggested to occur as a result of the animal's reduced immune response following the viral infection [16].

In addition to the effects of extrinsic factors on disease, intrinsic (host-specific) factors may correlate with disease prevalence. Host body size is often positively related to parasitic infection, perhaps because large hosts ingest more, increasing the chances of endoparasitism [17]. Larger hosts also present a greater surface area which increases the likelihood of ectoparasite attachment [18]. Alternatively, sex biases in infection can occur which may be related to differences in behaviour and/or reproductive strategies between the sexes [19], [20]. Intrasexual variation in infection may be related to individual differences in reproductive investment with those investing more in reproduction becoming infected more often [19], [20]. Males might also have a reduced immune function associated with increased levels of testosterone [21], [22]; however, infection is not always male-biased [20]. Morphologically, size of the testes is a useful proxy for high testosterone levels, aggression, and territoriality [23]–[25]; hence this feature may be linked to the risk of infection. Other morphological features of interest include zygomatic arch width (ZAW), which is suggested to relate to dominance rank and reproductive investment in both sexes [26], [27]. Dominant individuals may be more susceptible to infection due to their increased activity [28] and, in some cases, due to the increased stress levels that they endure [29]. Finally, spleen size may correlate with infection because of its role in lymphocyte production [30]. However, the situation is complex because animals with large spleens may, in fact, be better-equipped to resist pathogens and consequently experience a lower prevalence of infection [31], [32].

The North American grey squirrel (*Sciurus carolinensis*) is a useful species with which to examine the links between morphology, parasitism, and disease. Grey squirrels play host to various parasites [33] but are also reservoirs for squirrelpox virus (SQPV), which causes a disease that is normally lethal to the native red squirrel (*Sciurus vulgaris*) [34], [35]. The aims of the current study were to determine the links between seroprevalence of SQPV in grey squirrels and various aspects of morphology and parasitism. Specifically, we determined whether individuals with higher parasite burdens were more likely to be exposed to SQPV; whether males in general and, specifically, whether those with larger testes were more likely to be seropositive for SQPV; and whether there were any links between spleen size, ZAW, and SQPV exposure.

## Materials and Methods

### Ethics statement

Squirrel carcasses were obtained from the Northern Ireland (N.I.) Forest Service, who granted permission for their use. Animals were culled according to methods outlined in “Control of Grey Squirrels for Red Squirrel Conservation” [36]. No individuals were obtained specifically for the purposes of this study.

### Specimen collection

**Figure 1:**
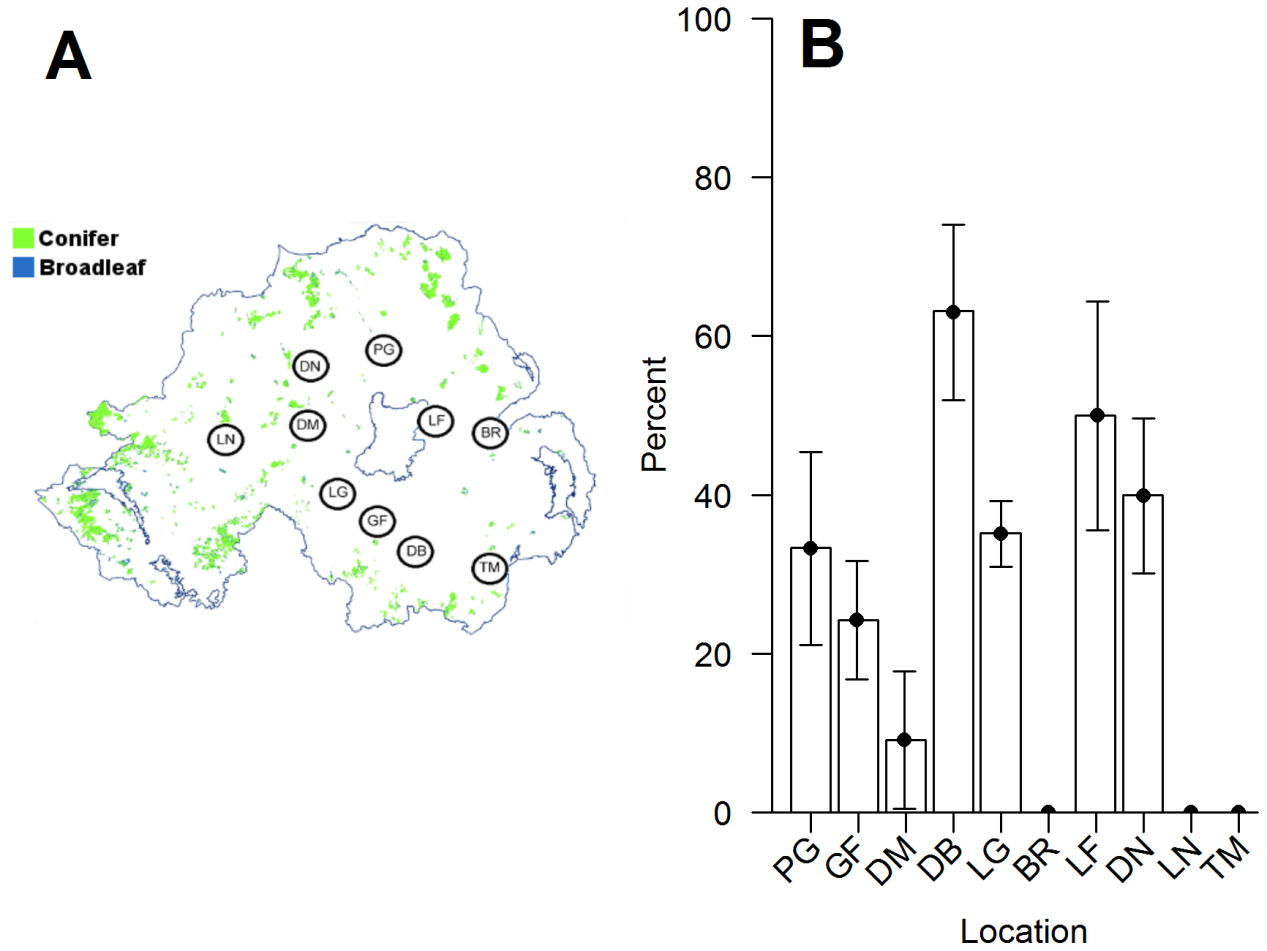
(A) Distribution of sampling locations across various forests in N.I. (B) Percentage of specimens (± S.E.) found to be positive for squirrelpox virus within each location. Forest locations are denoted as: PG – Portglenone; GF – Gosford; DM – Drum Manor; DB – Drumbanagher; LG – Loughgall; BR – Belvoir; LF – Larchfield; DN – Derrynoyd; LN – Lissan; TM – Tollymore.

Grey squirrel specimens were collected as part of a government forestry culling programme, from various sites across N.I. (February 2008–February 2009 inclusive). Samples were available during all four seasons (spring, summer, autumn, and winter). The locations, sample sizes, and years that culling of grey squirrels began were: Belvoir (n = 10) (2001); Larchfield (n = 12) (no data); Lissan (n = 11) (2008); Tollymore (n = 1) (1998); Drum Manor (n = 12) (2000); Portglenone (n = 15) (2002); Loughgall (n = 119) (1992); Gosford (n = 43) (1988); Drumbanagher (n = 19) (1993); and Derrynoyd (n = 51) (2001) (Fig. 1). Specimens were stored at −20°C until processed.

### Morphometric measurements

Following ectoparasite removal (see below), each specimen was sexed and weighed (±1 g), and the ZAW was measured using Vernier callipers (±1 mm). The body length of each specimen was measured from the base of tail to the tip of the nose (±1 mm). Specimens were then dissected to remove the spleens and testes, which were subsequently weighed (±0.001 g; Ohaus Explorer, Ohaus Corporation, Pine Brook, NJ, USA). Organs were placed in an oven at 60°C and dried to constant mass before being weighed again. A 1-ml blood sample was taken from each specimen for SQPV exposure analysis (see below). The stomach and intestines were removed for endoparasite analysis (see below).

### Determination of parasite burden

The head, tail, dorsal and ventral sides of each specimen were combed five times in each direction using a flea comb (Dimensions: Length = 9 cm; Width = 5.2 cm). Ectoparasites were removed and stored in 70% ethanol. Ectoparasites were examined at ×15 magnification under a Kyowa microscope (model SDZ-PL, Hashimoto 3-chrome, Sagamihara, Kanagawa, Japan) and identified using key morphological features (e.g. ctenidial combs) [37]. In each squirrel specimen, the stomach was separated from the intestine. The contents were removed and liquefied in 0.9% (m/v) saline solution. Stomachs and intestines were then examined and washed with saline to ensure no parasites were attached to the walls of either organ. Endoparasite burden was determined by examining the contents of the stomach and intestine of each specimen at ×15 magnification under a Kyowa microscope (Kyowa SDZ-PL, Hashimoto 3-chrome, Sagamihara, Kanagawa, Japan). Any parasites found were identified from morphological features (e.g. the presence and structure of bursa or spicules in males, or measurements from the tail to the vulva in females). The McMaster technique was used to estimate coccidian parasite burden as described previously (see [33]). Coccidia were quantified as follows: 0 resulted in a negative recording (−); one to three oocysts per field was recorded as (+); between three and four, (++); between five and fifteen, (+++), and over fifteen as (++++) [33].

### Determination of SQPV exposure

The presence of antibodies against SQPV was determined from blood samples for all specimens using an ELISA [35]. The presence of antibodies was used as a measure of SQPV exposure as it is difficult to replicate the virus in high titres. This provides a measure of viral exposure at the time of culling. An optical density reading of >0.2 at 492 nm was taken as the cut-off to differentiate specific antibody positive serum samples from negative serum samples.

### Statistical analyses

Data analyses were carried out in “R” version 2.15.2 [38]. Data distributions for all variables were significantly different from normal (Kolmogorov-Smirnov test, p<0.001). Season, sex, and location differences in SQPV seroprevalence were compared using Fisher's exact tests. Variation in SQPV exposure was examined according to the length of time that grey squirrel colonies were established in each location to determine whether seropositivity within a location changed with time [39]. The date that culling was first carried out by N.I. Forest Service was used as a proxy for the establishment of grey squirrels in a given area.

Generalized Linear Models with a binomial distribution and log link function were used to assess the potential relationships between SQPV seroprevalence, parasite load, and morphological features. Minimal models were selected based on comparisons of Akaike Information Criteria (AIC) [40]. First, any collinearity between morphological features was examined using Spearman's rank correlations to determine if any organ masses could be omitted from models. No collinearity was observed (p>0.05 in all cases) so the associations between each morphological feature (body mass, ZAW, mass of the testes, and spleen mass) and SQPV exposure were examined by entering each feature singly as an explanatory variable in individual models with SQPV exposure as a response variable. In the event of a variable holding an explanatory power of p<0.1, that variable was then entered into a single model comprising the main effects and two-way interactions between morphological features (except mass of the testes) on SQPV exposure (Model A, Table 1). This model was then expanded to include the main effects and two-way interactions between morphological features and various forms of parasitism (nematode, coccidia, and ectoparasites) on SQPV exposure in a single model (Model B, Table 1). The final models examined the combined effects of morphology, parasitism, and sex on exposure to SQPV, these were: The main effects and two-way interactions between morphology and sex on SQPV exposure (Model C1, Table 1) and the main effects and two-way interactions between parasitism and sex on SQPV exposure (Model C2, Table 1).

**Table 1:** Statistical models.

Model	Cohort	Maximal model	Minimal model	Sig. effects	z	P
A	All	BM+ZAW+SPL+BM*ZAW+BM*SPL+ZAW*SPL	BM+ZAW+SPL+BM*ZAW+ZAW*SPL	BM	2.47	0.014
B	All	BM+ZAW+SPL+COX+NEM+ECT+BM*ZAW+BM*SPL+BM*COX+BM*NEM+BM*ECT+ZAW*SPL+ZAW*COX+ZAW*NEM+ZAW*ECT+SPL*COX+SPL*NEM+SPL*ECT+C*NEM+COX*ECT+NEM*ECT	BM+SPL+ZAW+NEM+COX+ZAW*SPL+ZAW*NEM+ZAW*COX	NS	NS	NS
C1	All	BM+SPL+ZAW+SEX+BM*SPL+BM*ZAW+BM*SEX+SPL*ZAW+SPL*SEX+ZAW*SEX	BM+SPL	BM	2.98	0.003
C2	All	COX+NEM+ECT+SEX+COX*NEM+COX*ECT+COX*SEX+NEM*ECT+NEM*SEX+ECT*SEX	NEM	NS	NS	NS
D	Males	BM+TES+ZAW+SPL+BM*TES+BM*ZAW+BM*SPL+TES*ZAW+TES*SPL+ZAW*SPL	BM+SPL+TES+BM*TES	SPL; TES	1.97; 3.38	0.049; <0.001
E	Males	BM+SPL+TES+ZAW+COX+BM*SPL+BM*TES+BM*ZAW+BM*COX+SPL*TES+SPL*ZAW+SPL*COX+TES*ZAW+TES*COX+ZAW*COX	BM+SPL+TES+ZAW+COX+BM*SPL+BM*TES+BM*COX+ZAW*COX	TES; COX; BM*TES; BM*COX	2.81; −2.31; −2.18; 2.30	0.005; 0.021; 0.030; 0.022
F	Males	BM+SPL+ZAW+NEM+BM*SPL+BM*ZAW+BM*NEM+SPL*ZAW+SPL*NEM+ZAW*NEM	BM+SPL+ZAW+NEM+BM*ZAW+SPL*ZAW+ZAW*NEM	ZAW; BM*ZAW; SPL*ZAW; ZAW*NEM	−2.67; −2.18; −3.01; 2.55	0.007; 0.029; 0.003; 0.011
G	Males	BM+SPL+TES+ZAW+ECT+BM*SPL+BM*TES+BM*ZAW+SPL*TES+SPL*ZAW+SPL*ECT TES*ZAW	BM+SPL+ZAW+TES+BM*SPL+BM*ZAW+SPL*ZAW	SPL*ZAW	−2.01	0.045

Statistical models used to determine the effects of various morphological features as well as parasitism and sex on SQPV exposure. All models used a binomial distribution and log link function. Abbreviations are denoted as follows: BM = Body mass, SPL = Spleen mass, TES = Mass of testes, ZAW = Zygomatic arch width, SEA = Season, COX = Coccidial burden, NEM = Nematode burden, ECT = Ectoparasite burden, SEX = Sex, “*” = interaction, NS = No significance (p>0.05 for all effects in minimal model).

The inclusion of mass of the testes as an explanatory variable excluded females from the analysis. Therefore, further models examined male specimens only. Initial analysis revealed that there was a significant seasonal variation in mass of the testes (Kruskal-Wallis: χ^2^ = 9.13, df = 3, p = 0.028). Hence the main effects of season, mass of the testes, and body mass, and the interactions between season and mass of the testes, as well as mass of the testes and body mass on SQPV exposure were included in a combined model. In this case, none of the terms showed a significant explanatory power (p>0.05). Therefore, the factor “season” was omitted from further models. The first model featuring data for just males included the main effects and two-way interactions of all morphological features (body mass, ZAW, mass of the testes, and spleen mass) on SQPV exposure (Model D, Table 1). Thereafter, additional models examined the interactions between morphology and the various types of parasitism (coccidia, nematodes, and ectoparasites) (Models E-G, Table 1).

## Results

Of the 236 grey squirrel specimens examined, 113 were female and 123 were male. Of these, 33.6% of females and 32.5% of males tested positive for the presence antibodies against SQPV. There was no significant difference between the proportion of males and females exposed to SQPV (χ^2^ = 0.002, df = 1, p = 0.96). The seroprevalence of SQPV varied with location (Fisher's exact test: p = 0.007) (Fig. 1A+B). At one site, 63.2% of the individuals tested positive for SQPV antibodies, whereas all other sites had seroprevalences ≤50% and three populations showed no evidence of exposure. There was no significant relationship between the percentage of seropositive individuals and the time since first culling in that location (least squares regression, F_1,7_ = 2.10, r^2^ = 0.231, p = 0.191). However, there was a significant difference between seroprevalence in winter (40.3% of individuals seropositive) compared to summer (21.7% of individuals seropositive) (χ^2^ = 4.12, df = 1, p = 0.042).

Specimens were infected with a variety of ecto- and endo- parasites (Table 2). Ectoparasites found included the flea, *Orchopaeus howardii*, the louse, *Neohaematophinus sciurinus*, the tick, *Ixodes ricinus*, and the mite, *Androlaelaps fahrenholzi*. The overall prevalence of ectoparasites was 70.3%. Endoparasite species identified included coccidia (*Eimeria* sp.) oocysts and nematodes, *Trypanoxyuris* (*Rodentoxyuris*) *sciuri*, *Trichostrongylus retortaeformis*, and *Trichuris* sp. The prevalence of coccidial parasites and nematodes was 70.3% and 53.7% respectively.

**Table 2:** Sex differences in morphology and parasitism.

	Males			Females			
Feature	N	Mean	S.E.	N	Mean	S.E.	P
Body mass (g)	151	528	8	130	545	8	0.28
ZAW (mm)	148	33.8	0.3	127	33.5	0.3	0.49
Spleen mass (g)	151	0.31	0.03	126	0.29	0.02	0.66
Mass of testes (g)	152	0.47	0.04	NA	NA	NA	NA
Ticks	127	0.06	0.02	105	0.01	0.01	0.036
Fleas	127	4.77	1.18	105	2.11	0.28	0.06
Mites	107	0.05	0.03	92	0.03	0.02	0.87
Lice	127	1.13	0.21	105	0.84	0.19	0.08
Total ectoparasites	127	5.99	1.21	105	2.98	0.36	0.06
Nematodes	151	3.83	0.59	130	6.08	0.92	0.10
Coccidial score	139	1*	0.1*	117	1*	0.2*	0.74

Mean, standard error of the mean (S.E.) (* denotes median and standard error of the median), and sample size (N) of various internal and external morphometrics (measured in grams (g) and millimetres (mm)), and ecto- and endo-parasite burdens in males and females. Also shown are the p-values (P) from Mann-Whitney U tests examining sex differences for each parameter. No sex differences were evident in any of these tests (p>0.05) except with tick burden where males exhibited higher burdens than females (p = 0.036).

### Effects of morphology and parasitism on SQPV exposure in all individuals

There were no sex-differences in body mass, spleen mass, or ZAW (Table 2). When the main effects and two-way interactions between morphological features (body mass, ZAW, and spleen mass) were considered in a single model, only body mass had a significant association with SQPV exposure, with larger individuals experiencing exposure more often than smaller individuals (Model A, Table 1). There were no significant interactions between any features in the minimal model. ZAW and spleen mass were not significantly related to SQPV exposure either (p>0.05). Similarly, when parasitism, morphology, and their interactions were considered, there were no significant relationships with SQPV exposure (p>0.05 in all cases) (Model B, Table 1). Body mass was the only variable which remained significant when the combined effects of morphology and sex on SQPV exposure were considered (Model C1, Table 1). Finally, no significant results were noted when parasitism and sex were examined in a model (Model C2, Table 1).

### Effects of morphology and parasitism on SQPV exposure in males

**Figure 2:**
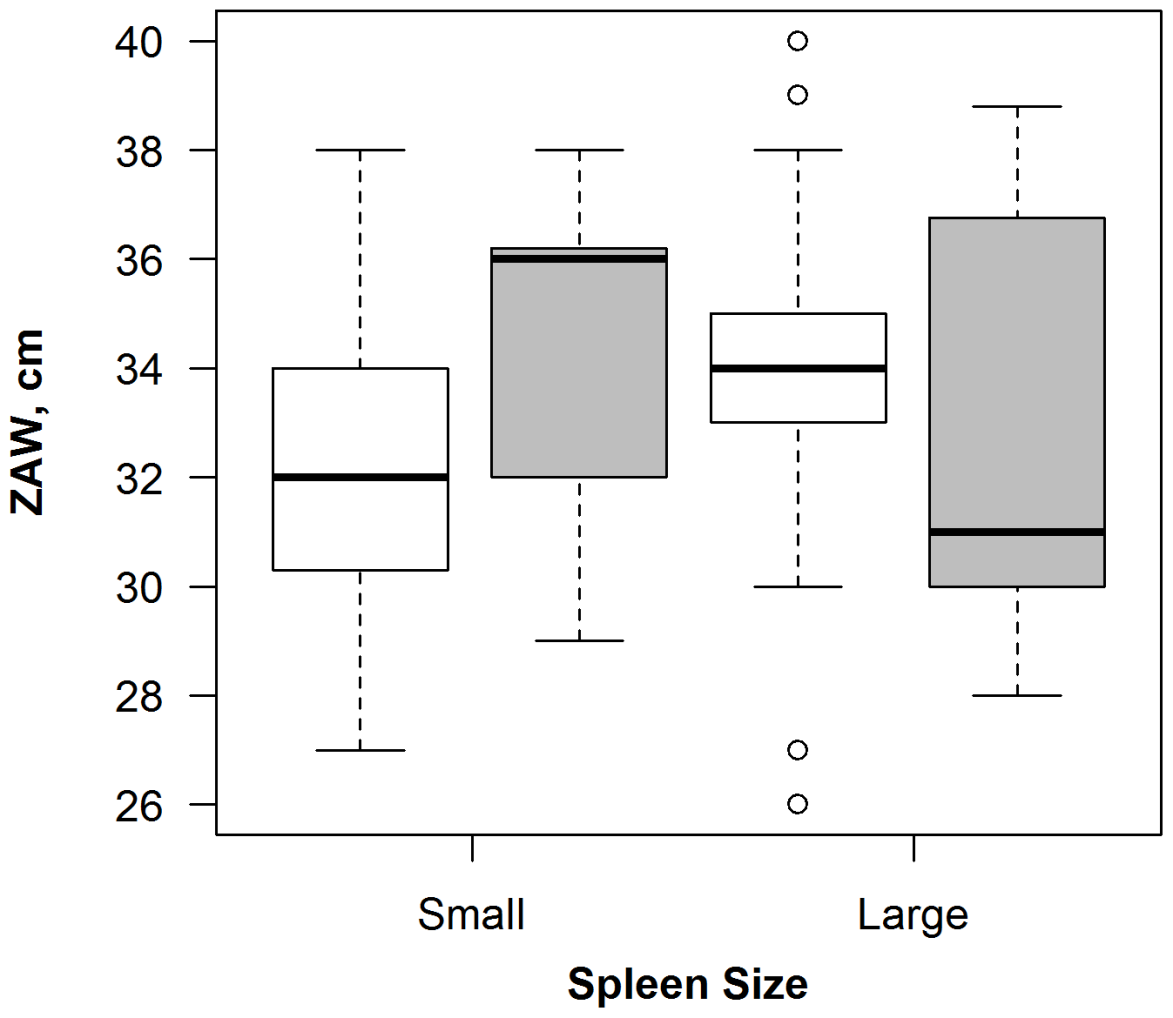
Effect of ZAW (cm), and spleen size (Small, Large) on prevalence of SQPV in males. Non-infected individuals are denoted by open bars, infected individuals by dark bars. Plot “whiskers” denote maximum and minimum values, and “box” shows upper and lower quartiles and the median. Outliers are shown as open circles and are defined as datapoints which lie outside the range of the upper quartile+1.5 times the interquartile range, or the lower quartile−1.5 times the interquartile range.

There were no significant relationships between body mass or ZAW and SQPV exposure (Model D). However, specimens with larger testes and those with larger spleens were more likely to have been exposed (Table 1). Examination of the associations between morphology and coccidial burden on SQPV exposure yielded significant interactions between parasite load and various morphological characteristics on the seropositivity of SQPV (Model E, Table 1). Large males with high coccidial burdens were more likely to be exposed to SQPV than smaller individuals with low coccidial burdens. In addition, large males with large testes were more likely to be exposed to SQPV than small individuals with small testes (Table 1). When morphology, nematode burden, and their interactions with SQPV exposure were considered (Model F, Table 1), a significant interaction was noted between spleen mass and ZAW on SQPV exposure; individuals with wide ZAW and small spleens were more likely to be SQPV seropositive than individuals with wide ZAW and large spleens (Fig. 2). In addition, small males with a narrow ZAW were more likely to be exposed to SQPV than larger males with wide ZAW. There was a significant interaction between ZAW and nematode burden; males with a wide ZAW and high nematode burdens were more likely to be exposed to SQPV than those with wide ZAW and low nematode burdens. Finally, when ectoparasites were considered, the interaction between spleen mass and ZAW noted previously was conserved (Model G, Table 1).

## Discussion

Invasive species have been cited as major causes of population extinctions in several animal and plant classes worldwide [41]. The North American grey squirrel has a major detrimental effect on native red squirrel populations in Britain and Ireland, in part because they are reservoir hosts for SQPV, which is deadly to red squirrels. Although some researchers have investigated the epizootiology of SQPV disease [42]–[44], less work has examined the various morphological, physiological, and environmental characteristics that might make individual grey squirrels more susceptible to, and potentially better able to transmit the virus to red squirrels [33], [45]. The current study investigated the putative relationships between morphology, parasite infection, and SQPV seroprevalence in grey squirrels, and documented any geographical and seasonal variation that was found in these characteristics. We found that larger individuals and males with larger testes were more likely to have been exposed to SQPV. This is consistent with predictions that larger, dominant individuals may experience increased exposure to infection, possibly by moving further distances and spending more time performing activities such as foraging which might expose them to pathogens [46]. When the additional effects of endo- and ecto-parasitism were considered, a high coccidial burden was found to be associated with increased SQPV exposure in larger individuals. It is unclear why this might be the case but coccidial infection is often present as a more serious, acute infection in young or juvenile animals, and more chronic in adults [47]. Thus, adults may appear to have an increased coccidial prevalence and perhaps this is then associated with an increased likelihood of SQPV seroprevalence.

There were sex differences in the relationships between morphology and parasitism, with many significant effects on SQPV exposure evident in males but not in the pooled data of males and females. For example, males with a wide ZAW and high nematode burdens were more likely to be SQPV seropositive, which suggests that older dominant males may be more likely to be exposed to SQPV. The fact that apparent correlates of dominance and reproductive activity are related to parasitism and exposure to SQPV suggests that there are costs to these traits, i.e. some individuals, often males, may increase infection risk in favour of reproductive success [19], [20], [48], [49]. Male squirrels are also known to be more aggressive, have large home ranges, fight amongst each other, and chase females during oestrus [49], [47]. These behaviours might increase exposure to SQPV. The fact that some forms of parasitism co-occur with SQPV exposure supports the contention that one pathogen may be exploiting the diversion of the host's immune response to the other [14], or that immunomodulation, either by the parasites or SQPV, can increase susceptibility to the other.

Spleen mass was an important indicator of SQPV exposure in males as those with larger spleens were more likely to have been exposed. When dominance was also considered, it was noted that males with a wide ZAW (potentially dominant individuals) with small spleens and males with a narrow ZAW (potentially subordinate individuals) with large spleens were more likely to have been exposed to SQPV (Fig. 2). It is unclear whether SQPV influences spleen size or *vice versa* but it would seem that there is a trade-off between being dominant and investing in immunity and that there is at least two modes of infection; one in which older, dominant individuals are exposed and another in which younger, subordinate animals are exposed. A potential explanation could be that individuals with small spleens are less well-equipped to deal with SQPV infection, especially if they have an increased risk of exposure to SQPV because they are reproductively active (as evidenced by wide ZAW and larger testes), or if they have elevated testosterone levels which may compromise immune system functionality (as indicated by larger testes) [21]. It is also possible that the relationship between mass of the testes and SQPV is a result of SQPV causing testicular swelling, which is characteristic of infection with other viruses such as mumps virus [50].

Concurrent with previous studies, the seroprevalence of SQPV varied according to location, with some areas apparently clear of the infection and other areas comprising large numbers of exposed animals [39]. While there was a possibility for the more established colonies from different areas to have greater percentage seroprevalence, this was not apparent. Geographical variation in SQPV seroprevalence might be a result of environmental features such as the distribution of suitable habitats [51] as well as the presence of other organisms which could act as reservoir hosts or vectors for SQPV, however, at present no other hosts have been identified [39]. Although there was no overall seasonal effect on SQPV seroprevalence, there was a significant difference between winter and summer exposure, with almost twice as many individuals testing seropositive during the winter. As winter is the main breeding season [52], reproductive males may become exposed to SQPV when they patrol areas in search of receptive females [46]. Alternatively, increased SQPV seroprevalence during the winter may be a consequence of close social contact between individuals when communal nesting increases [53], or when individuals cluster around food resources [54].

## Conclusions

Despite various conservation efforts, including culling grey squirrels [55], and reintroduction and supplementary feeding of red squirrels [54], the number of grey squirrels in the U.K. and Ireland continues to rise whilst the number of red squirrels falls. This study investigated the potential aspects of SQPV seroprevalence, which may contribute to this trend. While there may be potential biases in data collection (e.g. young or naïve individuals may be trapped more easily), the sample size was large enough to obtain representative animals spanning different cohorts within the population. Spatial and temporal variation in SQPV antibodies was evident, with notable increases during the winter. Reasons for this remain unclear but it may well be a result of individuals being in closer contact with each other either as a result of mating behaviours, feeding and/or nesting. Of note is that larger males with larger testes and those with concurrent nematode or coccidial infections were more likely to be exposed to SQPV. In addition, subordinate males with large spleens were also more likely to be exposed (Fig. 2). Hence, SQPV transmission may occur when animals are large and dominant, and potentially already under stress from other infections and also when they are young and small. Finally, individuals with larger spleens were more likely to experience SQPV exposure, suggesting that spleen size may increase as a response to infection. It is hoped that natural immunity may develop in red squirrel populations, as has occurred in European rabbits presenting resistance to the myxoma virus [16]. It may therefore be prudent to encourage the expansion of those red squirrel populations which have already been exposed to SQPV and survived, perhaps by supplementary feeding, coupled with the culling programmes which are already in place for grey squirrels, to promote the development of natural immunity within the population.
